# Disability and innovation in virtual production: Towards an inclusive framework for accessible filmmaking

**DOI:** 10.1177/13548565251410685

**Published:** 2026-01-08

**Authors:** Jessica Hoare, Rhys Miles Thomas, Paul Burke, Sally Lisk-Lewis, Greg Mothersdale

**Affiliations:** 1 2112 Cardiff University, School of Journalism, Media and Culture, Cardiff, UK; 2 Independent Researchers; 3 145351 University of South Wales, Faculty of Business and Creative Industries, Cardiff, UK

**Keywords:** virtual production, inclusive, accessibility, d/Deaf/Disabled/Neurodivergent (DDN), film production, television production, diversity, equity

## Abstract

The increasing adoption of Virtual Production (VP) technologies in TV and film production may have the potential to offer expanded inclusivity, greater creativity, logistical advantages, and improved sustainability. This paper explores such claims in the context of diversity and inclusion, and the increasing attention being paid to issues of accessibility and inclusivity for d/Deaf, Disabled, and Neurodivergent (DDN) cast and crew within TV and film production. The paper presents a case study of *We Dream of Nothing* and *Flying Without Wings*, two test shoots that developed and implemented accessible VP working practices, addressing the access requirements of DDN cast and crew. These productions were conducted through cycles of co-developed participatory action research, as part of an industry–academia R&D collaboration, in which accessibility interventions were planned, implemented, observed, and reflected on collaboratively. Drawing on a case study, the paper proposes an emergent Inclusive Virtual Production Framework for accessibility, integrating inclusive practice across all stages of production, from pre-production and recruitment to on-set workflow. It documents a developing model of accessibility intervention in which the affordances of VP facilitate DDN inclusion centred on dignity, respect, and collaboration. The paper highlights practices that enable DDN talent and leadership on set, detailing the work and accessibility practices tested and developed across the two productions. Situated within broader debates of screen sector workforce inequality and equity, this paper addresses the long-standing underrepresentation of DDN individuals in both creative and technical roles. Despite many initiatives which have promoted diversity targets, truly inclusive working practices are yet to become standardised on set. In response, this paper proposes working practices that enable DDN inclusivity. As part of its knowledge equity approach, the paper offers first-person reflections on the process and the challenges from the project team. Finally, we summarise both the challenges and the significant opportunities presented by VP technologies to create thriving, equitable production environments.

## Introduction

Despite ongoing growth, the creative industries’ workforce continues to be shaped by long-standing patterns of privilege and exclusion (Brook et al., 2015). In particular, the TV and film sector, despite its significant contribution to the global economy, suffers from homogeneity across the workforce and fails to reflect the diversity of society (Wreyford, O’Brien, and Dent 2021). Within UK statutory frameworks, such as the Equality Act 2010, employers are required to provide legal protection against workplace discrimination and to make reasonable adjustments for disabled individuals who may be placed at a substantial disadvantage compared to non-disabled colleagues. The question of how to approach these adjustments in a meaningful and sustainable way is central to ensuring long-term change within the TV and film industry. The affordances of virtual production (VP) may offer new opportunities for creating accessible and inclusive working environments. However, limited research exists on the potential of VP to support a greater range of d/Deaf, Disabled, and Neurodivergent (DDN) access requirements. To our knowledge, no prior published studies have directly addressed DDN adaptation within VP; therefore, this paper represents the first attempt to do so.

The study is approached through participatory action research, underpinned by knowledge equity principles, embedded within a research and development (R&D) project led by two production companies, GlassShot working in collaboration with Turbulence. Centred on the use of on-set virtual production, a production technology where LED panels enable video or computer-generated images to be displayed as a backdrop in real-time. The project aims to create and test production practices that were inclusive to a wide range of cast and crew. The project was supported by Media Cymru, a media sector research, development and innovation programme led by the Centre for the Creative Economy at Cardiff University. Running from 2022 to 2026, the programme aimed to deliver equitable benefits, such as job creation and increased GVA within the local economy, by offering competitive industry R&D funding interventions, skills development, and collaborations between academia and the creative industries. Across the programme, funding is allocated to projects that support inclusion and diversity across the creative industries.

## Disabled labour in TV and film production

Inequality in the media and screen industries has been widely documented with a range of discrimination and inequality being indicated (Conor et al. 2015; Eikhof and Warhurst 2013; Gill 2013; Hesmondhalgh and Saha 2013; Nwonka 2020; Nwonka and Malik 2018; O’brien 2014; Randle and Hardy 2016; Wing-Fai et al. 2015). In the UK, an estimated 24% of the working-age population is disabled, while recent figures suggest just 9.7% of screen industries workers are disabled (Collins 2024; Powell 2024). Data on workforce disability disclosure in screen industries are highly fragmented, owing to differences between methodologies, definitions, and scope, that hinder comparisons. Changing approaches to disability and impairment further problematise historical data, some authors discuss disability through the lens of physical impairment and a deficit model, whereas more recent reports often adopt the social model of disability and some further expand the category by including questions about neurodiversity (Randle, K., Wing-Fai, L., & Kurian, J. 2007; Taylor 2020, 2022; UK Screen Alliance 2019). Despite the differences in data, there are indicators that disability disclosure is on the rise in the media sector. In 2023, Ofcom reported that 10% of television workers disclosed a disability up from 6% in 2019 (Ofcom 2019, 2023).

Beyond the limits of available data, qualitative research highlights how disabled workers experience TV and film labour in practice. Studies have shown that workers are often concerned that disclosing a disability could obstruct their career development (Randle, K., Wing-Fai, L., & Kurian, J., 2007). When they do disclose, DDN workers have reported that working in television production, especially with established broadcasters (such as BBC and Channel 4), provided more favourable conditions for workers with disabilities. In contrast, the pace and the resource allocation of independent companies mean that they are less likely to accommodate disabled workers (Randle and Hardy, 2016). UK trade union body, Bectu, found that 65% of disabled screen industry professionals feel insecure about their jobs, and over half of disabled screen workers are currently out of work (Bectu 2024).

Roulstone and Williams’ research indicated that disabled workers also experience significant obstacles in vertical and horizontal mobility, and a ‘glass partition’, meaning that career progression and opportunity to work outside of DDN related content were limited. These issues were partially attributed to the lack of management awareness and mentoring, where gaps exist in the appropriate understanding and implementation of reasonable adjustments, as well as a lack of disabled leaders and mentors to support disabled workers’ career development (Roulstone and Williams 2014). Horizontal barriers were found to keep disabled talent working within disabled related content with further studies demonstrating that disabled workers are often assigned to work on disability-specific programming and remain siloed in that type of production (Randle, K., Wing-Fai, L., & Kurian, J., 2007; Randle and Hardy, 2016). Wilson-Kovac’s investigation of the experience of the disabled workforce indicated that while there was often attention to physical accessibility there existed a lack of attention to other more impervious, often social or attitudinal, barriers (Wilson-Kovacs et al., 2008).

## Inclusion, belonging and the social model of disability

In recent years, efforts to support diversity in the TV and film industries have proliferated with many broadcasters and regulators making increased commitments to representation and inclusion (BBC 2024; Channel 2025; ITV 2024; Ofcom 2023; ScreenSkills 2023). Major commissioners and funders have adopted and (crucially) report on diversity targets (BBC 2024; BFI 2020; Channel 2025). In the UK, a key initiative working towards inclusive, systemic change is The TV Access Project (TAP) (ScreenSkills 2023). Launched in 2022 to drive structural change in the TV and film industry and ensure full inclusion of disabled talent by 2030, TAP is a collaboration between 10 major broadcasters and streamers committed to removing systemic barriers and creating long-term accessibility solutions. By fostering collaboration between disabled-led organisations and the mainstream industry, TAP aims to eliminate barriers and create equitable opportunities for disabled professionals across all levels of TV production.

These are welcome initiatives and should be recognised as vital contributions to the ongoing work of improving disability inclusion in TV and film production. Addressing the historical underrepresentation of DDN talent, both in front of and behind the camera, requires a commitment to challenging industry norms that obstruct inclusive and accessible working practices. Despite industry-wide discussions of equity and commitments to inclusivity, disabled professionals continue to face significant barriers to entry, career progression, and on-set accessibility (Ansell 2021; J. Collins 2024). Funder and commissioner level initiatives are an important step. We contend, however, that targets and standards often focus on the demographics of who is involved in a production rather than addressing the fundamental question of their experience and how to create inclusive working environments. This may lead to a scenario where diversity targets are met on paper, but the working conditions on set remain inaccessible to many disabled professionals.

Achieving meaningful inclusivity requires a shift beyond numerical targets to a deeper transformation of industry practices. Here, we draw on the concept of ‘belonging’ to inform our approach to inclusion (Collins and Liebelt 2021; May, 2013; Shaikh and Haider 2024; Wolbring and Nguyen 2023). We contend that a key component in this approach is the recognition that inclusivity is not merely a statistical goal but a dynamic, relational process of shifting power relations and that such work requires continual, committed labour, a willingness to be uncomfortable, stepping back from defensiveness, and the capacity to try doing things differently (Wise 2022). Within this context, screen industries R&D funding has an important contribution to make in de-risking the experimentation required to support the development of DDN accessible working practices both with regards to the use of new technologies (such as VP) but also supporting on-set culture change. This paper draws on the significant body of scholarship that focuses on inequality in the UK creative industries, we most closely align with those who highlight the differences between inclusion and belonging and demonstrate the need to engage critically with both, if diversity work is to be effective (Dovey and Bhajam 2023; Malik 2023; Wreyford, O’Brien, and Dent 2021). Informed by lived experience of disability across the project team and supported by the literature indicated above, the authors acknowledge that diversity initiatives have an important role to play in increasing representation, but argue that creating DDN belonging in work environments requires a deeper cultural shift across a sector, with greater emphasis on creating a culture that not only prioritises equity in process and mindset but actively expects to change and be positively shaped by equitable processes and approaches.

The integration of accessibility practices within TV and film production is often framed in terms of time-based, logistical and financial constraints; however, many of the most significant barriers are social and cultural (Ansell 2021). Too frequently, an immediate focus on cost and logistics overlooks the fact that accessibility is not merely about accommodation, but an essential component of both inclusive and socially responsible culture and labour practice. Arguments against accessibility based on cost often serve as a convenient excuse and disregard the deeper need for a cultural shift in how productions approach and plan for disability inclusion. We also note the claims made by Heumann and Joiner that the financial argument has been used perpetually throughout the history of disability activism to avoid affording disabled communities their human rights (Heumann and Joiner 2020). Efficiency may be a fiscal obligation, but it is not a statutory one, whereas in the UK compliance with the Equality Act 2010 requires employers to make reasonable adjustments to avoid discrimination. In this context, the claim that efficiency must come first sustains discrimination. Elsewhere, Foster & Wass have skilfully demonstrated that such calls for efficiency are underpinned by post-Fordist models of production, where the desired efficiency is achieved through precarious, over-flexible labour and the assumption of a worker unencumbered by care, health, or access needs (2013).

Cost is but one part of the work associated with creating accessible TV and film productions and is secondary in complexity to the broader challenge of reconfiguring how disability is recognised, or overlooked, across a diverse range of spaces. As Titchkosky argues, the way access is regarded is deeply tied to how disability appears, or fails to appear, in the situations we navigate (Titchkosky, 2011). Accessibility issues relate to facilities and infrastructure but are also endemic in workplace cultures. Disabled-led reviews of screen industry labour have highlighted colleagues’ attitudes and a lack of understanding as persistent barriers (Barr, G., Lubran, H., Thorne, J. and Player, K. 2023). In response, this paper approaches the issues of disabled labour from the perspective of the social model of disability. Widely adopted across UK policy and embedded within the discourse of creative industries organisations, the model rejects the idea that conditions disable individuals but argues that society and environmental conditions disable people (Oliver 1990). This approach foregrounds the relational and systemic dimensions through which disability is produced and navigated within society.

## Accessibility and the affordances of virtual production

Against these structural and cultural barriers, virtual production offers specific affordances that can support greater accessibility for DDN cast and crew, particularly by decentralising production and reducing logistical constraints. Conventional production is often shaped by location shoots, long travel times, and extended working hours, conditions that can disadvantage or exclude disabled workers (Swords et al., 2022). Virtual production enables the recreation of real-world locations, not only does this provide access to locations that may be inaccessible to many DDN cast and crew – it also reduces the need for physical travel and enables greater scheduling flexibility. Beyond the logistical benefits, VP provides a controlled environment where access interventions can be more readily made. Studios can be designed or adapted to include quiet spaces, accessible toilets, and other key DDN infrastructure. Additionally, the technical affordances allow for precise management of lighting, sound, and environmental factors, particularly valuable for individuals with sensory sensitivities or mobility impairments.

Across the project team, there was significant knowledge and experience of the barriers DDN individuals face on set. Table 1 collates the issues the team identified at the start of the project by drawing on our research and professional knowledge. The barriers detailed reflect physical, sensory and cultural components of DDN experience. Through research into universal design and neurodiversity in creative workspaces, we included interventions such as quiet spaces, sensory accommodations, clear communication protocols, and structured working days (CIPD 2018; Steinfeld and Jordana, 2012). We also addressed communication and cultural challenges, recognising that exclusionary behaviours and the absence of structured disclosure processes are well-documented issues for disabled workers (Fevre et al., 2013). The barriers identified are organised into four areas of focus: awareness and training, planning and logistics, physical and sensory environments, and communication and culture, and summarised in Table 1.

**Table 1.** Identified barriers to inclusive and accessible virtual production practice.

Focus	Barrier	Potential solutions
Recruitment & hiring	Disabled talent often excluded at the point of recruitment due to informal hiring networks, lack of accessible job postings, or fear of disclosure	Use DDN networks and organisations to connect with talent. Clearly communicate commitments to access and accessible recruitment practices in recruitment materials and include access coordinators in hiring and onboarding
Awareness & training	Lack of awareness and understanding of disability awareness and inclusion among wider cast and crew	Implement disability inclusion training; engage access consultants with lived experience. Provide additional role-specific training and ensure accessibility is embedded in all onboarding processes
Planning & logistics	Insufficient pre-production planning and allocation of resources for accessibility	Integrate accessibility into budgeting and scheduling from the outset via access coordinators
	Demanding location shots and inflexible production schedules that don’t accommodate different access needs	Use VP to build flexibility into schedules; allow for staggered call times or remote options where possible
	Insufficient breaks and rest periods built into shooting schedules	Ensure set breaks are adhered to; consult with disabled cast/crew to assess individual needs around rest
	Lack of orientation and familiarisation opportunities for cast/crew	Offer in-person orientation; include pre-shoot visual stories in pre-production documents shared with cast and crew
Physical & sensory environment	Inaccessible physical production environments and locations	Conduct accessibility audits of all locations; ensure safe drop off zones and suitable parking, prioritise step-free access, clear signage, wide door frames and corridors, accessible bathrooms, availability of quiet spaces
	Limited availability of quiet spaces and sensory accommodations on set	Designate and clearly label quiet zones, provide noise-cancelling tools, and sensory aids
	Inadequate catering options to meet diverse dietary requirements	Consult with cast/crew and catering teams on dietary needs well in advance; offer clearly labelled options
Communication & culture	Lack of clear communication about access requirements and adaptations on set	Create an accessibility onboarding process that supports DDN disclosure; assign an access coordinator to manage communication
	Exclusionary on-set culture and behaviours (e.g. shouting, inappropriate comments)	Promote inclusive workplace policies; establish and enforce a respectful code of conduct

## Case study: Accessible virtual production in practice

### Methodology

This study adopted a participatory action research approach, in which industry and academia worked together to identify problems, understand existing evidence and test interventions. In line with the principles of action research, the project followed an iterative cycle of planning, action, observation, reflection, iteration, with each cycle generating both practical improvements and new knowledge. This approach was an appropriate choice as it effectively bridged academic and industry expertise, allowing for productive collaboration.

Crucially, we grounded our collaboration in the principles of knowledge equity, meaning that the lived experience and professional expertise of collaborators, producers, directors, and access coordinators were treated as equal in value to academic perspectives (Glazer 1998; Hall and Tandon 2017). Rather than positioning practitioners as research subjects, the project aligns with co-production scholarship that foregrounds collaborators as co-authors and equal partners in knowledge creation (Miles et al. 2022). This is reflected in the authorship of this paper. Accordingly, the practitioner reflections presented later in this paper are not treated as supplementary data, but as knowledge contributions in their own right, reflecting the principle that lived experience and professional expertise generate valid forms of knowledge. Their inclusion demonstrates how accessibility was enacted and experienced, and they sit within the main text to inform and substantiate the case study.

This commitment to co-production was woven through the design of the research process. An initial feasibility study explored VP’s potential for creating greater accessibility on-set. A subsequent larger funding award supported two test shoots, *We Dream of Nothing* and *Flying Without Wings*, these served as iterative cycles of action research. Across the two shoots, access requirements were identified and addressed through multiple mechanisms:

• Access passports captured individual requirements at the point of recruitment and informed production planning.
• Studio access audits identified environmental and logistical barriers prior to shooting.
• Field notes and on-set observations.
• Project team reflections captured further aspects of lived experience, creative perspectives, and critical insights into the impact on workflows.

In the following sections, we discuss the pre-production research and planning, the importance of setting expectations regarding on-set behaviour, and highlight the key, set-wide strategies that fostered greater inclusivity throughout the shoots.

## Pre-production research and planning: Embedding person-centred approach to production

The directing and producing team worked with an Access Coordinator from the start of pre-production. Access Coordinators are responsible for overseeing and facilitating inclusion, access, and work adjustments for cast and crew members working on set. They increase accessibility on set by collaborating with individuals and Heads of Production to identify solutions for removing barriers to access and inclusion. These professionals enable DDN cast and crew to deliver their best work whilst maintaining their physical and mental health. In the UK, Access Coordinators implement an ‘access first’ approach, applying the Social Model of Disability. 

The project team worked with DDN cast and crew to identify the adjustments that would enable them to work most effectively. This process was largely led by the individuals requiring access support, and further facilitated through consultation with the Access Coordinator, whose role was not to impose solutions but to enable dialogue, advise on best practice, and propose specific adaptations where needed. This ensured that interventions addressed real barriers while remaining feasible within production constraints. Examples included implementing structured break times, support with travel, providing disability awareness training for all cast and crew, and normalising inclusive practices such as quiet spaces, and collaborative communication protocols.

At recruitment, all cast and crew were offered the chance to complete accessibility forms, based on the access passport model. An access passport is a live record of adjustments that outlines an individual’s specific requirements and accommodations required to perform their job effectively and with dignity. By offering these forms to all cast and crew the process was normalised, and the production team were able to identify and address barriers at individual and group level. The Access Coordinator managed the administration of access passports initially. Once they had been completed, access requirements were assessed in partnership with the Co-Producer/Directors and factored into the shoot planning, budget, and studio choice. An access audit was conducted of the studios to ensure they could meet the requirements of all cast and crew. In addition to accessibility forms, all cast and crew were provided access to online training in disability, access and inclusion in the film and TV sector at recruitment.

**Figure 1.**
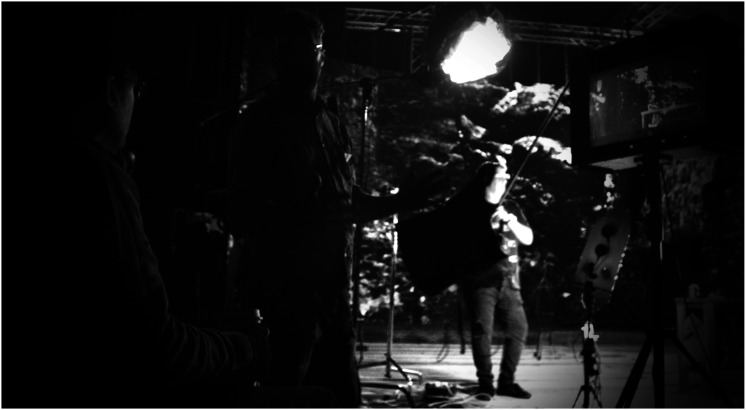
Rhys Miles Thomas and Paul Burke, Co-authors, Co-producers, and Co-directors on the VP set of ‘We Dream of Nothing’.

A VP studio was selected for each shoot and all filming was entirely conducted within these locations and backdrops provided in VP (Figure 1). No external locations were used. Once on set, the Access Coordinator and the First Assistant Director worked closely together to ensure a smooth production process. The Access Coordinator focused on managing individual access requirements, while both worked together to manage scheduling and keep to fixed break times throughout the shoot.

## Set and setting culture

On set, the importance of open communication, a positive working environment, and the prioritisation of wellbeing was explicitly reinforced at the start, during breaks, and at the end of each shooting day. The Co-Producer/Directors and Access Coordinator led all cast and crew briefings. These briefings covered key areas including behaviour, preferred language and access requirements, as well as outlining the planned workflow, introduced break times that were kept to throughout the shoot, and reiterated the commitment to access across the team. This approach aimed to ensure that accessibility and inclusivity were embedded in the production culture and to support those that needed further adjustment or changes to their access requirements to feel safe in speaking up. To reinforce these values, the Co-Producer/Directors and Access Coordinator led by example, actively promoting respectful and supportive interactions. This approach required a shift from traditional on-set behaviours, explicitly establishing the set as a space free from shouting and exclusionary ‘banter’. The expectations for behaviour on set were introduced and communicated to all members of the team ahead of the shoot and as part of the start of day briefings. While simple these steps sought to create a DDN friendly environment and aimed to foster a considerate and accommodating work environment for everyone on set. For DDN individuals, these behavioural guidelines sought to establish a space where individuals felt comfortable have expressed, and continually expressing, their access requirements.

Additional measures were introduced to create a working environment that was both accessible and supportive of well-being. Each of these working practices was developed directly in response to access requirements identified in the access passports. They included:

**Travel, Flexible Schedules and Tours:** Many disabled participants identified travel as a barrier to participation. Arrangements were made to provide accessible vehicles where needed, ensuring that all individuals could access the set with ease. Staggered start and leave times supported those cast and crew who needed more time for travel or shorter days.
**Orientation Tours and Name Labels:** The Access Coordinator led individual tours on arrival to familiarise cast and crew with the studio layout, facilities, and check in regarding access requirements. Name labels were provided for everyone on set, this aimed to reduce the cognitive load associated with remembering names, particularly beneficial for neurodivergent and learning-disabled participants, and to encourage more inclusive communication across the team.
**Quiet Spaces and Sensory Accommodations:** Regular rest breaks and access to quiet areas were repeatedly requested in access forms. Dedicated quiet spaces were established to offer individuals a place to recharge when needed. Prior notice was given of any loud noises on set. Additional accommodations, such as sensory-friendly areas, noise-cancelling headphones and personalised sensory equipment, were made available based on individual requirements communicated via the access passports completed during recruitment.
**Ongoing Monitoring and Adjustments:** Regular check-ins were conducted throughout production to identify and resolve emerging issues. Cast and crew were encouraged to share feedback to ensure the production remained aligned with its accessibility commitments. Monitoring by the Access Coordinator ensured measures remained effective. This ongoing dialogue also prompted additional DDN disclosures during the shoots, as some participants initially hesitated to share access needs for fear of being a ‘burden’ or ‘difficult’. These late disclosures required a few last-minute adjustments but underscored the importance of sustained communication and trust.
**Dietary and Catering Requirements:** Access requirements also routinely featured specific allergies, phobias, sensory issues, diet plans and condition-related food requirements. This required the access coordinator to plan thoroughly and work very closely with the catering service to accommodate these requests.
**Accessible Scheduling:** Informed by the repeated requests for scheduled rest breaks identified during recruitment, pre-production planning prioritised energy management and sustainable working. Fixed break times ensured that individuals had ample time to rest and recharge. Slightly shorter working days were introduced, where necessary, to accommodate varying requirements. Any proposed extensions, such as an additional 30 minutes of shooting, were agreed through consultation with those affected by the extension.

Collectively, these simple but effective practices demonstrate that increasing accessibility and a culture of DDN inclusion does not always require complex or costly solutions. In the case of our pilot project, it was contingent on understanding evidence, building access processes into recruitment as standard, pre-shoot planning, clear communication, and a willingness to adapt. Crucially, the nature of virtual production, with its flexibility and stable location made it easier to plan, adjust quickly, and accommodate individual needs in real-time. The virtual production studios allowed for a more controlled environment, ideal for managing sensory sensitivities, reducing physical strain, and maintaining consistency across the shoot.

## Accessibility challenges and adaptive responses

The project revealed a range of challenges, many of which were resolved through relatively simple adjustments (see Table 2). Environmental issues included a cold studio, mitigated by providing space heaters, blankets and encouraging warm clothing; and lighting that interfered with lip-reading, addressed through visual cues. Logistical challenges emerged around time, with some DDN participants requesting longer familiarisation periods. Cultural challenges also surfaced, such as initial anxieties from non-DDN cast and crew about using the wrong language, which were met by fostering an open environment where mistakes could be acknowledged and repaired.

**Table 2.** Post-shoot reflections on inclusive and accessible virtual production practice.

Focus	Barrier identified	Response/Solution implemented	Lessons learned/Implications
Physical/Environmental	Cold VP studio without heating	Provided heaters, blankets, encouraged warm clothing	Have additional heaters available and offer them repeatedly. Encourage cast and crew to bring warm clothing
	Cable ramps are bulky and can block wheelchair access	Incorporate the taping down of cables into the standard workflow even when regularly moving camera equipment wherever possible. Clearly communicate when access routes may be temporarily blocked	Even with cable ramps and core access routes in place, this still required continual monitoring
	Lighting made lip-reading difficult	Introduced visual cues and repositioned speakers	VP environments must be audited during shooting for sensory accessibility
Process/Logistical	Insufficient time for familiarisation on-set	Allocated extra rehearsal/walkthrough time in the second shoot	Additional time around arrival is critical for accessibility
Cultural/Communication	Anxiety around ‘saying the wrong thing’	Normalised mistakes, encouraged apology/repair	Fostering an open learning culture for non-DDN cast and crew is vital
	Hesitancy to disclose conditions	Supportive, inclusive environment encouraged openness and led to further disability disclosures during the shoot	Inclusive culture may enable further disclosure – this requires a degree of on-set flexibility
	Need for mutual trust between disabled and non-disabled colleagues	Emphasised empathy, patience, two-way exchange	Accessibility is relational, not just procedural

## Practitioner reflections

After the test shoots wrapped, the project team reflected on the processes, outcomes, and their perceived impact. These accounts, authored by the Co-Director/Producers, Access Coordinator, R&D Producer, foreground the situated expertise of the industry professionals directly involved in delivery of this study. In line with our knowledge equity approach, these accounts are not treated as data, but as a core part of the co-created knowledge presented in this co-authored paper.

## Rhys Miles Thomas

Back in my early twenties, before my disability changed things, I was acting in an American TV remake of Jason and the Argonauts. We filmed in Turkey, and one of the fight scenes took place on a mountaintop accessible only by helicopter. 20 years later, now a wheelchair user, returning to that mountain would be impossible. Yet I still want to get back up there and fight that dragon again. At first, this project was about finding a way back to that mountain.

This project was never about ticking boxes or meeting quotas. It was about enabling talented people to do exceptional work when given the right environment. The successes were numerous: a learning-disabled trainee with crowd anxiety described it as his ‘best day ever’; a wheelchair user, long accustomed to barriers, finally felt free to shine; a disabled VP artist realised creative visions he had only imagined before; learning-disabled actors performed unburdened by the usual constraints; and a neurodivergent crew member, with years of prior experience, felt comfortable disclosing her condition for the first time. Across the two shoots we achieved 60% and then 70% DDN representation, and an experienced Deaf actor thrived in the open, supportive atmosphere.

## Paul Burke

For over two decades I’ve worked across film, television, and advertising, always striving to create safe, collaborative spaces for cast and crew. Yet it was only through conversations with Rhys about Virtual Production that I began to see how new technologies could unlock something more radical: genuine accessibility. What started as a conversation quickly became an experiment in reimagining how production could work.

We built the shoots around a simple principle: centre the person, not the process. That meant ensuring meaningful DDN representation across cast and crew and adjusting the workflow to support people rather than forcing people to adapt to workflow. VP still required the usual preparation and testing, and we faced the same time pressures as any production, but VP allowed us to respond more flexibly. When things did not go to plan, we treated those moments not as failures but as opportunities to adapt, learn, and carry the team forward together.

What stood out most for me was the atmosphere we created. Everyone was thanked personally, paid promptly, and recognised as vital to the process. Accessibility wasn’t treated as an add-on but as part of the creative and human fabric of the production. Looking back, I see this as a model for how the industry could operate more broadly: combining technical innovation with a culture of care, so that people are able to do their best work.

## Sally Lisk-Lewis

As an access coordinator, my role is about anticipating and asking about everyone’s access requirements ahead of our days in studio. Two recurrent requests on the accessibility forms included scheduling in regular rest breaks and time for familiarisation with the environment in rehearsal and on the shoot days. The VP environment itself, a newly opened studio with many accessibility features already in place, made the assessment and subsequent adjustment process easier, less time-consuming, less stressful, and reduced costs.

Planning was essential. Because we were working in a single VP space for each shoot, we could focus our efforts on delivering the right adjustments quickly. Chill-out and seating areas proved vital, especially during the second shoot with 10 actors with additional learning needs, but in practice they benefited everyone. We also fostered an atmosphere where mistakes could be acknowledged and corrected without stigma. Accessibility is always a two-way exchange, built on trust, empathy, and patience between those with lived experience and those still learning. We took the time to make it clear to everybody on the productions, that there are no silly questions, and that it’s ok to make mistakes sometimes, be it with language or how we approach a certain situation. We encouraged the team to acknowledge the error and apologise for it. Accessibility must be a two-way exchange, based on trust, patience, empathy and understanding, between those with lived experience and those still learning to understand why it matters.

## Greg Mothersdale

In my roles across industry and academia, I have been involved in numerous Virtual Production R&D projects. These experiences consistently show me the benefits of VP: the ability to create content in real-time, make faster decisions on set, and plan shoots with a level of control not possible on location, where weather, transport, and facilities often create barriers.

From an R&D perspective, this was an opportunity to support a team determined to reshape production practice around inclusion. The team were able to experiment, source equipment and expertise, and establish new methods that actively increased DDN involvement at every stage of production. What began as technical development evolved into something deeper: a recognition that accessibility is as much about culture and collaboration as it is about technology. Studio infrastructure, technical capacity, and skilled crew all mattered, but the real progress came from open communication, shared problem-solving, and embedding accessibility into every decision. These projects not only demonstrated what was possible in two test shoots but also generated a body of knowledge and expertise that can inform future productions. In that sense, they represent a step toward systemic change in the screen industry.

## Towards an Inclusive Virtual Production Framework

The case study presented above demonstrates that inclusive virtual production rests on four interdependent principles: access-first planning, co-creation with DDN practitioners, flexibility in working processes, and ongoing monitoring and reflection. Together, these principles form the basis of our Initial work on an Inclusive Virtual Production Framework and emphasise that accessibility is not simply about accommodating individual needs but about cultivating a culture in which DDN practitioners can thrive. These principles translate into practical action across production stages. In pre-production, access-first planning means actively recruiting DDN talent, anticipating requirements through onboarding calls, access passports, and studio access audits. In production, co-creation and flexibility are embedded through orientation tours, inclusive briefings, name labels, quiet spaces, and adjusted scheduling, supported by regular check-ins and the coordinating role of the Access Coordinator. In post-production, structured reflection captures insights to refine future practice and share learning with the wider industry. The findings from this case study offer foundational principles for embedding accessibility within virtual production and an emergent framework that others can adapt, build on, and evolve in their own VP contexts. The outcomes of applying this approach during the test shoots were significant. Both shoots achieved high levels of DDN representation, ranging from 60 to 70% across the two productions. Trust built by the pre-production and on-set processes led to increased disclosure of access needs during shoots. Participants reported improved well-being and a greater sense of belonging, while the production itself demonstrated that increased control offered by the VP environment supported a greater number of access interventions.

## Conclusion

Our intention, in contributing to discourse on the social and technical implications of VP, is to demonstrate how the unique affordances of VP technology can be leveraged to create more inclusive and accessible production environments. In response, this paper presents tested interventions that support DDN labour and equity within VP. The action research undertaken has demonstrated that VP may address many of the accessibility challenges faced by disabled professionals in the screen sector. As the TV and film industries continue to embrace VP, it becomes increasingly important to consider how new technologies can enhance production and serve as a catalyst for broader systemic change within the industry. VP has the potential to address the long-standing underrepresentation of DDN talent both in front of and behind the camera. This case study highlights the potential of VP to democratise access for DDN talent, presenting the interventions and challenges involved in creating accessible VP shoots. As the sector continues to engage with the transformative potential of Virtual Production (VP), it is essential that this technological integration is underpinned by a genuine commitment to accessibility. This requires not only adapting existing practices but also actively rethinking and challenging on set cultures. By prioritising inclusive approaches, the industry has the potential to cultivate an environment in which creativity and equity both flourish.

While this study is primarily concerned with equitable labour and accessibility in film and television production, we acknowledge recent research that has highlighted the role of XR technologies in non-pharmacological therapeutic contexts, for example, Dance Movement Therapy (Radanliev 2024a, 2024b). These applications lie outside the scope of our work, they share an underlying concern with how new forms of media and media production can foster inclusion and well-being. Together, they underscore the broader potential of new media technologies to contribute to more inclusive cultural and social practices for disabled people. At the same time, they highlight the importance of foregrounding disabled creativity, showing how increased access to such technologies can enable creativity and support both well-being and labour market inclusion. Accessible working practices should not be seen as a limitation but as a means of unlocking new creative possibilities. When accessibility is prioritised, productions benefit from a wider pool of talent and diverse perspectives. VP, with its inherent adaptability, offers powerful tools for advancing these aims.

Finally, while this case study was developed through two test shoots, its broader applicability requires further validation across a range of production contexts. Further research is needed to examine how these practices translate across different budgets, genres, and working cultures. As the industry continues to embrace VP, we claim that it is key that accessibility remains at the forefront of these developments. VP is not only a tool for artistic and technical innovation but also a potential catalyst for systemic change. While inclusion in TV and film production must be understood not only through the technological affordances of specific innovations but also through broader cultural and attitudinal shifts, we have demonstrated that Virtual Production does support increased DDN participation in screen industries labour and has a significant role to play in broadening the range of people who can sustain and thrive in careers in the screen industries.
